# Molecular Diagnosis of Cetacean Morbillivirus in Beaked Whales Stranded in the Canary Islands (1999–2017)

**DOI:** 10.3390/vetsci9030121

**Published:** 2022-03-07

**Authors:** Idaira Felipe-Jiménez, Antonio Fernández, Manuel Arbelo, Simone Segura-Göthlin, Ana Colom-Rivero, Cristian M. Suárez-Santana, Jesús De La Fuente, Eva Sierra

**Affiliations:** Atlantic Cetacean Research Center, Veterinary Histology and Pathology, Institute of Animal Health (IUSA), Veterinary School, University of Las Palmas de Gran Canaria (ULPGC), Trasmontaña, s/n, 35413 Arucas, Spain; idaira.felipe101@alu.ulpgc.es (I.F.-J.); manuel.arbelo@ulpgc.es (M.A.); simone.segura101@alu.ulpgc.es (S.S.-G.); ana.colom101@alu.ulpgc.es (A.C.-R.); cristian.suarez@ulpgc.es (C.M.S.-S.); jesus.delafuente@ulpgc.es (J.D.L.F.); eva.sierra@ulpgc.es (E.S.)

**Keywords:** cetaceans, morbillivirus, beaked whales, Canary Islands, PCR, *Ziphius cavirostris*, *Ziphiidae*

## Abstract

A retrospective survey for detecting the cetacean morbillivirus (CeMV) was carried out in beaked whales (BWs) stranded in the Canary Islands (1999–2017). CeMV is responsible for causing worldwide epizootic events with the highest mass die-offs in cetaceans, although the epidemic status of the Canarian Archipelago seems to be that of an endemic situation. A total of 319 tissue samples from 55 BWs (35 Cuvier’s BWs and 20 specimens belonging to the *Mesoplodon* genus) were subjected to the amplification of a fragment of the fusion protein (F) and/or phosphoprotein (P) genes of CeMV by means of one or more of three polymerase chain reactions (PCR). RNA integrity could not be demonstrated in samples from 11 animals. Positivity (dolphin morbillivirus strain (DMV)) was detected in the skin sample of only a subadult male Cuvier’s BW stranded in 2002, being the earliest confirmed occurrence of DMV in the Cuvier’s BW species. The obtained P gene sequence showed the closest relationship with other DMVs detected in a striped dolphin stranded in the Canary Islands in the same year. A phylogenetic analysis supports a previous hypothesis of a cross-species infection and the existence of the circulation of endemic DMV strains in the Atlantic Ocean similar to those later detected in the North-East Atlantic, the Mediterranean Sea and the South-West Pacific.

## 1. Introduction

The cetacean morbillivirus (CeMV; genus *Morbillivirus*, *Paramyxoviridae* family, order *Mononegavirales*), consisting of a single linear molecule of negative-sense single-stranded RNA, has been responsible for major epizootic diseases in cetaceans, causing many of the biggest mass die-offs worldwide in these species [1]. The main pathological findings described in infected cetaceans are broncho-interstitial pneumonia, lymphoid depletion and nonsuppurative meningoencephalitis, as well as an increased susceptibility to opportunistic infections [1,2,3,4,5]. According to the stage of the infection, four presentation forms of the disease have been recently described [1]: acute and subacute systemic diseases, chronic systemic infections and chronic localized CeMV encephalitis.

Two lineages of CeMV have been proposed [1,6]: CeMV-1 for the “old” northern hemisphere lineage that includes dolphin morbillivirus (DMV) [7], porpoise morbillivirus (PMV) [7], pilot-whale morbillivirus (PWMV) [8] and beaked-whale morbillivirus (BWMV) [9] strains; and CeMV-2 for the “new” southern hemisphere lineage, consisting of virus strains detected in a Guiana dolphin (*Sotalia guianensis*) from Brazil [10] and in Indo-Pacific bottlenose dolphins (*Tursiops aduncus*) from Western Australia [11]. In addition, it has been recently reported that a novel morbillivirus was detected in a Fraser’s dolphin (*Lagenodelphis hosei*) stranded in Hawaii that is dissimilar to the BWMV previously identified from Hawaii and to other CeMV strains, showing an 83.9–88.7% nucleotide similarity depending on the P or N gens of these reported sequences [12].

Few cases of CeMV infections have been previously reported in beaked whales (BWs). The infection (BWMV strain) was first documented in a Longman’s BW (*Indopacetus pacificus*) stranded in Hawaii in 2010 [9,13]. A lymphoplasmacytic (nonsuppurative) primarily cerebral encephalitis co-infection with the herpesvirus was observed in that case. The same strain has also been detected in Hawaii in two BWs; in a Cuvier’s BW (*Ziphius cavirostris*) and in a Blainville’s BW (*Mesoplodon densirostris*) stranded in 2008 and 2010, respectively [13]. Pathological descriptions were not available for those two cases. The infection (DMV strain) has also been recognized in one of the seven Longman’s BWs that stranded together in Southern New Caledonia in 2013 [14], with no pathological descriptions; and in a Cuvier’s BW stranded in Italy in 2015 [15] with mild pathological findings in its lungs. More details (multifocal fibrinous bronco-pneumonia with mild, multifocal necrotizing bronchiolitis) were revealed upon a microscopic examination and associated with verocytotoxic (VT1) *Escherichia coli*.

Beaked whales are deep-diving marine mammals, a condition that predisposes them to suffer from decompression sickness (DCS) associated with the employ of mid-frequency sonar during military operations [16,17,18,19,20,21,22,23]. Several mass stranding events of BWs have occurred in the Canary Islands related to naval exercises, although there have been no more since the Spanish government imposed a moratorium on naval exercises in these waters in 2004 [24,25]. However, these species also face other anthropogenic threats, such as entanglements in or ingestions of marine litter and ship strikes [26,27,28]. They are also vulnerable to infectious diseases, such as in the described cases of verminous arteritis by *Crassicauda* spp. [29], brucellosis [30,31], the herpesvirus infection [9,32,33,34,35] and *Flavobacterium ceti* septicaemia [36].

The Canary Islands are located in a strategically geographic region within the Atlantic Ocean, in which the presence of at least six species from the *Ziphiidae* family has been recorded (https://www.canariasconservacion.org/Zifios-Ziphiidae.htm/ (accessed on 29 June 2021)) [37]. The aim of this study was to realize a retrospective survey on BWs stranded in the Canary Islands in order to determine the presence of CeMV in this subset of the population. Moreover, a phylogenetic analysis was performed in order to analyse the relationships between the obtained sequences with others available in these and other species.

## 2. Materials and Methods

The availability of the number of animals for this study was possible thanks to the permission for the management of stranded cetaceans granted by the Spanish Ministry of the Environment. Moreover, no animal was sacrificed and no experiments were performed with live animals, so ethical review and approval were waived.

**Figure 1:**
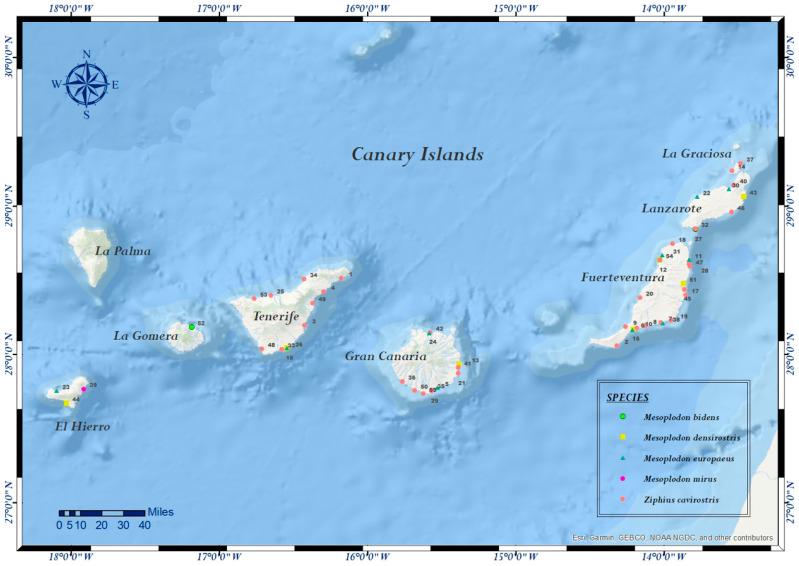
Map of the geographical distribution of beaked whales stranded in the Canary Islands between 1997 to 2017. Stranding sites for each beaked whale is identified by its case number. The species are represented with different colours.

Fifty-five BWs stranded along the coasts of the Canary Islands from November 1999 to May 2017 were included in this study, from which 35 specimens were Cuvier’s BWs and 20 specimens belonged to the *Mesoplodon* genus: one True’s BW (*Mesoplodon mirus*), two Sowerby’s BWs (*Mesoplodon bidens*), seven Blainville’s BWs and ten Gervais’ BWs (*Mesoplodon europaeus*). A map indicating stranding location of each animal (indicated by its case number and species) was created by the software ArcMap [38] and is shown in Figure 1. The information about each stranding (date, location with coordinates and type) and decomposition stage (grade 1: extremely fresh carcass; grade 2: fresh carcass; grade 3: moderate decomposition; grade 4: advanced decomposition and grade 5: mummified or skeletal remains) [39] is compiled in Table 1, as well as life history data (species, age category, sex and body condition). The nutritional status was classified as good, moderate, poor or emaciated in consonance with the anatomical parameters, such as the observable presence of marked bony process and prominent bones through the skin (the transverse and spinous vertebral processes and ribs), the observable presence of dorso-axial muscular mass and the presence or distribution of fatty tissue in several organs, taking into consideration the species and the age of the animal [22,25]. A complete postmortem examination, following standardized necropsy protocols [39,40], was performed on all of the animals from the study. Collected samples were fixed in 10% neutral buffered formalin solution, embedded in paraffin blocks, sectioned at 5 µm, stained with haematoxylin and eosin (HE) and examined under a light microscope. All the cases included in the present study were diagnosed during routine pathological and cause-of-death analyses of stranded cetaceans at the Division of Histology and Animal Pathology of the Institute for Animal Health (IUSA), Veterinary School, Universidad de Las Palmas de Gran Canaria. Immunohistochemistry was performed in molecular positive samples following a previously described standardized protocol. The tissue sections were incubated with a mouse monoclonal antibody against the nucleoprotein antigen of canine distemper virus (CDV, 1:200 dilution; CDV-NP MAb, VMRD Inc.), for which cross-reactivity with CeMV has been previously reported [41]. Positive controls included laryngeal tonsil from a CeMV-positive striped dolphin stranded in the Canary Islands in 2019. Virological analyses were performed in kept frozen samples (−80 °C).

According to the availability in each case, a total of 319 tissue samples were analyzed: the skin (50/319; 15.67%), lung (49/319; 15.36%), liver (47/319; 14.73%), kidney (46/319; 14.42%), brain (38/319; 11.91%), spleen (29/319; 9.09%), mesenteric lymph node (24/319; 7.52%), skeletal muscle (18/319; 5.64%;), intestine (6/319; 1.88%;), prescapular lymph node (4/319; 1.25%), mediastinal lymph node (2/319; 0.63%), thyroid gland (1/319; 0.31%), thymus (1/319; 0.31%), palate (1/319; 0.31%), oesophagus (1/319; 0.31%), penis (1/319; 0.31%) and blood (1/319; 0.31%). This information is available in Table 1.

Frozen kept samples were thawed and mechanically macerated for a subsequent simultaneous extraction of DNA and RNA by means of a QuickGene R Mini 80 nucleic acid isolation instrument with the DNA Tissue Kit S (QuickGene, Kurabo, Japan) according to the manufacturer’s instructions with some modifications: an RNA carrier (Applied Biosystems^TM^, Thermo Fisher Scientific Waltham, MA, USA) was added during the lysis step, as previously published [45].

Molecular detection of CeMV was performed using one or more of three polymerase chain reaction (PCR) methods. (1) was a modified conventional one-step reverse transcription polymerase chain reaction (RT-PCR), which amplifies a fragment of 426 base pairs (bp) from a conserved region of the phosphoprotein (P) gene. Primers and PCR protocol used were the following: ((DMV C: 5′-ATGTTTATGATCACAGCGGT-3′/DMV P2: 5′-ATTGGGTTGCACCACTTGTC-3′) and (94 °C × 4′–45 × (1′ × 94 °C−1′ × 51 °C–1′ × 72 °C)–7′ × 72 °C)). The obtained amplicons were analyzed by means of a 2% agarose gel horizontal electrophoresis [42,43,44]. (2) was a real-time one-step reverse transcription polymerase chain reaction (RT-qPCR) amplifying a size region of 192-bp from the fusion protein (F) gene using the following primers: (DMVFuF: 5′-GGCACCATAATTAGCCAGGA-3′/DMVFuR: 5′-GCCCAGATTTGTGCCTACAT-3′) and the PCR protocol (30′ × 48 °C–95 °C × 3′–40 × (95 °C × 3″–60°C × 30″)) [45] and (3) was a PAN RT-qPCR method based on SYBRN^®^ green dye [46] that successfully detects GDMV, PWMV and DMV strains. The primer set (Forward PAN-F (5′-CCTCTAACAGGGGATCT(A/G)CTC-′3) and Reverse PAN-R (5′-CCTGTGCCCTTTTTAATGGA-′3)) amplifies 205 bp from a region of the phosphoprotein (P) gene. The PCR protocol used was as follows: 50 °C × 10′–95 °C × 1′–40 × (95 °C × 10″–60 °C × 30″). The information about which PCR method was used for the detection of CeMV in each animal is specified in Table 1. A negative control (non-template) and an amplification-positive control (known cetacean morbillivirus RNA previously obtained in our laboratory) were added in each ADN/ARN extraction and PCR protocols described above. Genomic DNA digestion was performed in the total RNA/DNA extractions followed by a second purification following the same protocol as previously described. RNA extractions were then subject to a one-step RT-qPCR that amplifies the housekeeping gene encoding glyceraldehyde-3-phosphate dehydrogenase (GAPDH), as previously described [47], in order to ensure the high quality of the RNA [48]. In addition, all the samples were checked for the presence of herpesvirus by means of a pan-herpesvirus conventional nested PCR based on the DNA polymerase gene [35,49].

Previous to the Sanger sequencing method, the PCR products were purified using Real Clean spin kit (REAL^®^, Durviz, S. L., Valencia, Spain). BLAST algorithm (www.ncbi.nlm.nih.gov/blast/Blast.cgi/ (accessed on 10 November 2021)) [50] was used to compare the obtained amplicons with other somewhat similar sequences published in GenBank. The sequences were aligned using ClustalW algorithm through software MEGA X [51]. A total of 102 CeMV nucleotide sequences based on the P gene were recovered from GenBank to construct the phylogenetic tree, where three canine distemper virus (CDV) sequences were used to root the phylogram as an outgroup. The phylogenetic tree was constructed using the Maximum Likelihood Method and the Kimura 2-parameter model with a discrete Gamma distribution to model the evolutionary rate differences among sites (5 categories (+G, parameter = 0.7797)). The Bootstrap method (500 replicates) was applied to assess the reliability of the tree.

A phylogenetic tree based on the F gene was also created, which was constructed using the Maximum Likelihood Method and the Tamura 3-parameter model with a discrete Gamma distribution to model the evolutionary rate differences among sites (5 categories (+G, parameter = 0.5319)). A Bootstrap test from 500 replicates was also implemented. This analysis involved 52 nucleotide sequences, where two Peste des petits ruminants virus (PPRV) and two Phocine distemper virus (PDV) sequences established the root of the tree.

The DMV sequences from P gen and F gen were identified from this study and were deposited in GenBank under accession nos. OM055653 and OM055654, respectively.

## 3. Results

The presence of CeMV was detected in one animal (1/55; 1.82%), a subadult male Cuvier’s BW stranded to death in Fuerteventura in September 2002 in a good state of preservation (code 2, CET 182). Positivity was achieved in the skin sample (1/319; 0.3%). All the tested tissue samples from this animal were negative for the herpesvirus infection [35].

This Cuvier’s BW was one of the 14 BWs (designed as BW-3) stranded on the beaches of Fuerteventura and Lanzarote islands on 24 September 2002, temporally associated with naval exercises involving acoustic (sonar) activities (manoeuvers called Neo-Tapon 2002) [19]. Macroscopically, no systemic, inflammatory or neoplastic processes were noted, although the carcass showed severe diffuse congestion and haemorrhages, especially around the acoustic jaw fat, ears, brain and kidneys. Gas bubble–associated lesions and fat embolism were observed in the vessels and parenchyma of vital organs in the histopathological study (DCS) [19]. No pathogens were identified in routine aerobic bacterial cultures of the brain, lungs and spleens from this specimen [19].

The housekeeping gene was not amplified in samples from 11 animals of the 55 analyzed in the present study (20%). The RNA from these negative samples was too degraded for causes determining causes due to descomposition stages or the time elapsed since it was taken. Thus, only 44 animals were considered validated for the CeMV molecular analyses.

### 3.1. Nucleotide Identity

Skin samples tested positive for CeMV by means of two real time PCRs: a PAN RT-qPCR (P gene), as indicated by means of a visualization of an amplification curve at the 33rd cycle and a post-amplification melting-curve of 81–82 °C and a RT-qPCR (F gene), visible at the 31st amplification cycle and a post-amplification melting curve of 80 °C. A further sequencing of the P gene fragment (164 bp excluding primers) revealed that it had a 100% similarity (100% query cover) to DMV detected in two striped dolphins: one stranded in 2007 on the north-eastern coast of Portugal (GenBank acc. no. KP835995) and another stranded in the Central-East Atlantic Ocean (Canary Islands) in 2002 (GenBank acc. no. KJ139451).

### 3.2. Phylogenetic Analyses

**Figure 2:**
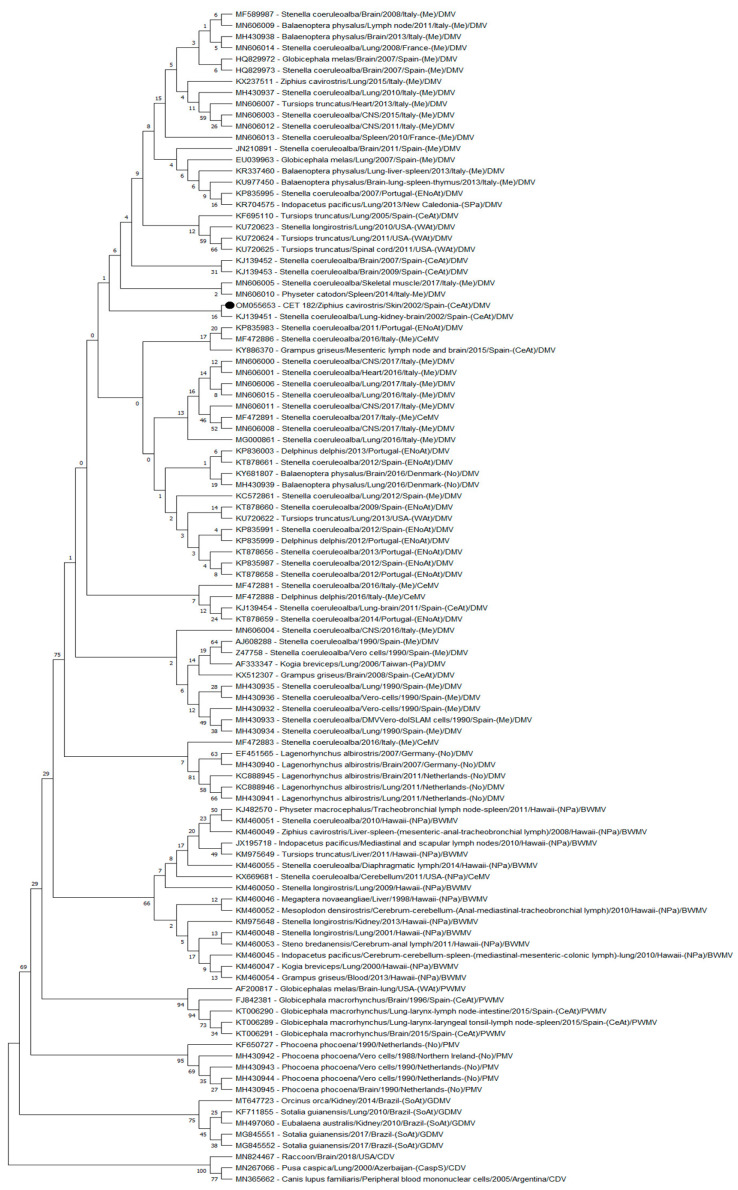
Maximum likelihood phylogenetic tree for the nucleotide sequences of the phosphoprotein (P) gene. The phylogenetic tree consists of 102 sequences from reported cases of cetacean morbillivirus. To construct the tree, were designed the Neighbour-Join and BioNJ algorithms along with the Kimura 2-parameter model and Gamma distribution to model the evolutionary rate differences among sites [5 categories (+G, parameter = 0.7797)]. The Bootstrap method was performed to resample 500 replicates and evaluate the reliability of the tree. The accession number from GenBank, the host, the sample of detection, the date of collection, and the geographic area of stranding were used to identify the nucleotide sequences. Abbreviations: ENoAt (Northeast Atlantic Ocean); WAt (West Atlantic Ocean); CeAt (Central Atlantic Ocean); SoAt (South Atlantic Ocean); Me (Mediterranean Sea); Pa (Pacific Ocean); NPa (North Pacific Ocean); No (North Sea); CaspS (Caspian Sea).

The nucleotide phylogenetic analysis based on the P gen showed that the obtained tree presents five main branches, where DMV, BWMV, PWMV, PMV and GDMV strains are clearly clustered in their respective clades (Figure 2). The DMV clade is supported by a Bootstrap value of 75 and contains a polyphyletic group of 71 sequences detected in 12 different species of odontocetes and mysticetes from the Mediterranean Sea (n = 39), the North-East Atlantic Ocean (n = 11), the Central-East Atlantic Ocean (n = 8), the North Sea (n = 7), the West Atlantic Ocean (n = 4) and the Pacific Ocean (n = 2) from 1990 until 2017.

Specifically, the first documented DMV sequences date from 1990 (n = 7), and since then, there are no more available sequences until 2002 (n = 1). From 2005 to 2017, there is at least one available sequence every year. Furthermore, from the phylogram analysis, we can observe two clearly separate clades within the DMV strain: one containing the sequences detected in the white-beaked dolphin species in the North Sea in 2007 and 2011 and in a striped dolphin stranded in Italy in 2016 and another larger clade containing two subclades.

In one of these, there are the sequences detected in striped dolphins during the first CeMV epizootic event in the Mediterranean Sea in the early 90s plus three sequences detected in a striped dolphin stranded in Italy in 2016 in a pygmy sperm whale (*Kogia breviceps*) stranded in the Pacific in 2006 and in a Risso’s dolphin (*Grampus griseus*) stranded in the Central-East Atlantic Ocean in 2008, respectively. In the other one, there are the rest of the DMV sequences detected until a date that includes the only two previous DMV sequences from BWs. The two DMV sequences previously detected in BWs are located in separate branches within the phylogram; the sequence detected in a Cuvier’s BW stranded in the Mediterranean Sea (Italy) in 2015 (GenBank acc. no. KX237511) is clustered with four sequences detected in three striped dolphins and one bottlenose dolphin (*Tursiops truncatus*) stranded on the coast of Italy in a period of five years (2010, 2011, 2013 and 2015, GenBank acc. nos. MH430937, MN606007, MN606003 and MN606012), and the sequence detected in a Longman’s BW in New Caledonia in 2013 is clustered with the sequence previously detected in a striped dolphin stranded in 2007 in the North-East Atlantic Ocean (Portugal, GenBank acc. no. KP835995), which is being shown to be 100% similar in a BLAST search with the sequence identified in our study. The isolated sequence obtained from this study was clustered with a single sequence, which was detected in a striped dolphin stranded in the Central-East Atlantic Ocean (Canary Islands) in 2002 (GenBank acc. no. KJ139451).

The obtained F gene fragment (125 bp excluding primers) had a 100% percentage of similarity (100% query cover) with 14 cetacean sequences (GenBank acc. nos. MT066174, MH430936, MH430935, MH430934, MH430933, MF589987, KU720625, KU720624, KU720623, MN606014, HQ829972, AJ608288, AJ224704 and Z30086). Most of these sequences (11/14; 78.6%) were detected in the Mediterranean Sea in the early 90s (8/14; 57.1%) and in the second Mediterranean epizootic event (3/14; 21.4%). The other three sequences (21.4%) were detected on the West Atlantic coast (USA) in 2010 and 2011.

**Figure 3:**
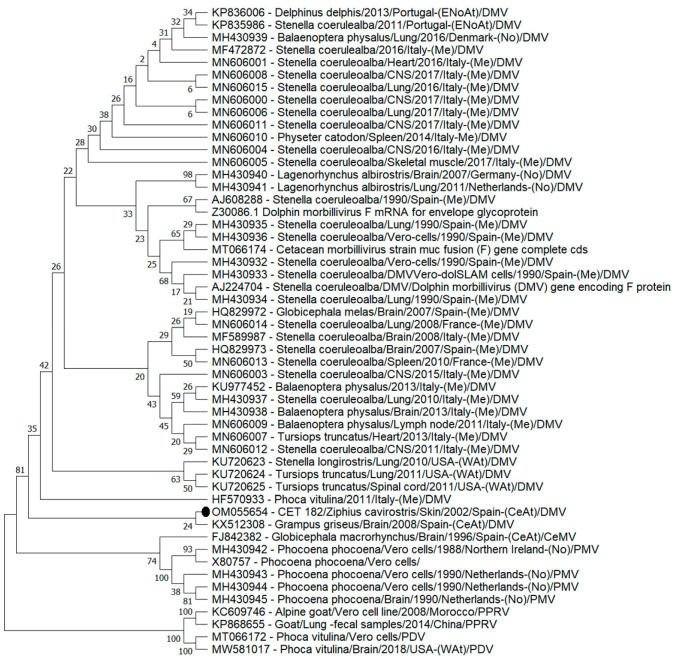
Maximum likelihood phylogenetic tree for the nucleotide sequences of the fusion protein (F) gene. The phylogenetic tree consisting of 52 sequences from reported cases of cetacean morbillivirus. To construct the tree, we designed the Neighbour-Join and BioNJ algorithms along with the Tamura 3-parameter model and Gamma distribution to model the evolutionary rate differences among sites (five categories (+G, parameter = 0.5319)). The Bootstrap method was performed to resample 500 replicates and evaluate the reliability of the tree. The accession number from GenBank, the host, the sample of detection, the date of collection and the geographic area of each stranding were used to identify the nucleotide sequences. Abbreviations: ENoAt (North-East Atlantic Ocean); WAt (West Atlantic Ocean); CeAt (Central Atlantic Ocean); Me (Mediterranean Sea); No (North Sea).

The phylogenetic nucleotide sequence analysis based on the F gene (Figure 3) showed a DMV clade supported by a Bootstrap value of 81 conformed by a polyphyletic group of 42 sequences detected in seven cetacean species and one harbour seal (*Phoca vitulina*). No other F gene sequences obtained from BWs are available in GenBank. The obtained BW sequence from this study was exclusively clustered with a sequence detected in a Risso’s dolphin (*Grampus griseus*) stranded in the Central-East Atlantic Ocean (Canary Islands) in 2008 (GenBank acc. no. KX512308).

### 3.3. Histopathology, Immunohistochemistry

**Figure 4:**
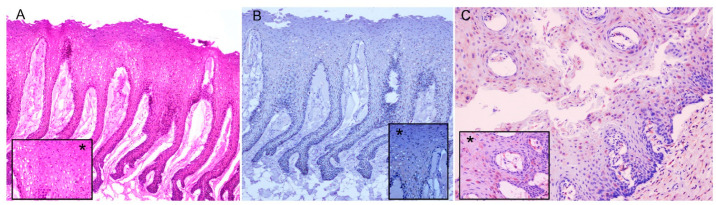
Histological and Immunohistochemistry evaluation of PCR-positive skin sample from a Cuvier’s Beaked whale (CET 182). (**A**) Histopathology. Most of the keratinocytes of the stratum spinosum exhibit ballooning (freeze artefact). HE stain, ×10. Inset: Detail of the ballooning (*). HE stain, ×40. (**B**) Immunohistochemistry. CDV immunostaining was not observed. Immunohistochemistry stain, X 10. Inset: detail of lack of specific immunostaining against CDV in keratinocytes (*). Immunohistochemistry stain, ×40. (**C**) Immunohistochemistry. Positive control for CDV antibody in laryngeal tonsil sample. Immunostaining was observed in the cytoplasm of epithelial cells. Immunohistochemistry for morbillivirus using an antibody to CDV nucleoprotein., ×20. Inset: detail of specific immunostaining against CDV in epithelial cells (*). ×60.

To date, only frozen skin tissue samples from this case remained available for the histopathological study. At the microscopic level, neither CeMV-associated lesions nor immunostainings for CDV were observed, although ballooning, interpreted as a freeze artefact, was present in most of the keratinocytes of the stratum spinosum (Figure 4A,B). The immunolabelling of CDV was observed in the epithelial cells from the positive control (laryngeal tonsil sample from a CeMV-positive striped dolphin stranded in the Canary Islands in 2019, Figure 4C).

## 4. Discussion

CeMV was detected in one of the 55 BWs analyzed for the presence of this pathogen in the Canary Islands. Specifically, the virus (DMV strain) was present in the skin sample of a subadult male Cuvier’s BW, representing the second molecular confirmation of a DMV infection in this species. The detection of DMV in a Cuvier’s BW was first reported in Italy in the lung sample of a male calf stranded in 2015 [15], 13 years after the sample from our study was collected. Therefore, the retrospective analysis of archived tissue samples carried out in our study exposed that this was the earliest confirmed occurrence of DMV in the Cuvier’s BW species.

Positivity was achieved in the skin sample by means of two real-time PCRs but not with the conventional one. The skin sample had high cycle-threshold values in both qPCRs and therefore low viral loads, which could be only detected with test methods with higher sensitivities when compared to more traditional assays [52]. CeMV has been previously detected in the skin samples of marine mammals with and without associated lesions [41,53,54]. No lesions were observed in the skin sample of the Cuvier’s BW from our study. In cetaceans, the effects of CeMV are widely variable from causing epidemics to subclinical infections. According to previous studies about CeMV in BWs, even if a study does not describe histopathological findings in detail, it seems that the infection does not cause severe pathological effects in these species [13,14,15]. The only exception is the first published case of a BWMV infection in a Longman’s BW stranded in Hawaii in 2010, which presented an associated nonsuppurative encephalitis but which was also co-infected with the herpesvirus [9]. In addition to the herpesvirus, secondary infections or co-infections with other viruses, bacteria, fungi, protozoans or parasites have been frequently described [2,48,55,56,57,58,59]. In our study, the presence of bacterial pathogens and a herpesvirus infection was ruled out [19,35].

The level of detection of CeMV in stranded BWs in the Canary Islands (1.82%; 1/55) in an 18-year period (1999–2017) is low compared with previous studies in other geographical regions, which range from 5.7% to 48.1% (Table 2). This variability in the level of detection is partially due to the fact that some of those retrospective studies included eligibility (inclusion) criteria or morbillivirus-related lesions, species classically considered DMV hosts, such as striped and bottlenose dolphins and/or areas and years in which some epizootic events or outbreaks were involved [1,48,60]. However, the absence of DMV detection in other samples from our study does not rule out the presence of DMV in such samples. The assessment of RNA integrity is a critical first step for detecting false-negative samples. Current epidemiological knowledge of CeMV in Canarian waters indicates the presence of some inter-epizootic sporadic endemic presentations of the infection with the detection of the virus in 1996, 2002, 2005, 2007–2009, 2011–2012, 2015 [44,59,61,62], 2016 and 2018–2020 (unpublished data). The exception is an unusual mortality event in 2015 involving short-finned pilot whales and the PWMV strain [63].

The isolate from this study is one of the only two earliest CeMV sequences available in GenBank detected after the first epizootic event on the Spanish Mediterranean coast in 1990–1992 [73], both of them detected in the same year in the Canary Islands in two different cetacean species and highly similar. The individual from our study stranded in 2002, and the obtained sequences showed the closest relationship with another DMV detected in cetaceans from the Canary Islands (with a striped dolphin stranded in the same year with a systemic chronic infection (GenBank acc. no. KJ139451) [61] according to the nucleotide sequence alignment and phylogenetic analysis of the P gene) and with a Risso’s dolphin stranded in 2008 (with chronic localized CeMV encephalitis (GenBank acc. no. KX512398) [66] according to the phylogenetic analysis of the F gene). This information seems to support a previous hypothesis of a cross-species infection [15,44,61,66,67,69,74,75]. The P gene nucleotide analysis also showed that the isolate is highly similar to one detected in a striped dolphin stranded on the North-East coast of Portugal in 2007 (GenBank acc. no. KP835995) [64] in the middle of the second epizootic event on the Spanish Mediterranean coast [55,76,77], which also clustered with samples from the Mediterranean Sea from 2007 to 2013. The F gene nucleotide analysis showed that very similar sequences were detected in the Mediterranean Sea in the first and second Mediterranean epizootic events. Taken together, these results are consistent with the previous supported idea that CeMV is endemic in the Central and North-East Atlantic and that interanimal transmissions might occur through the Strait of Gibraltar. This viral exchange could cause Mediterranean outbreaks and unusual mortality events in that region due to the relatively high density of animals, specifically striped dolphins, and their gregarious behaviour [57,61,64,76,78,79,80,81,82,83,84,85]. In addition, the P gene sequence from Portugal (GenBank acc. no. KP835995) is clustered with the DMV isolate detected in a Longman’s BW from New Caledonia (GenBank acc. no. KR704575) as previous studies have indicated [64]. Thus, both DMV sequences detected in BWs from the Central East Atlantic in 2002 and the South-West Pacific in 2013, respectively, show high similarities to the sequence detected in a striped dolphin from the coast of Portugal in 2007, which is also similar to sequences from the Mediterranean from 2007 to 2013. It has been previously reported that undefined cetacean species, due to their extensive patterns of migration and their interactions with certain dolphin populations, may mediate the trans-oceanic spread of CeMV, driving the dispersal and evolution of this virus [72,75,86]. The Cuvier’s BW species has a worldwide distribution, featured in almost all temperate, sub-tropical, tropical, sub-polar and polar waters [87]. Moreover, this species presents migrating behaviours in the Atlantic Ocean, and it could probably act as a reservoir spreading the disease into more susceptible populations as previously proposed for fin whales [75,88,89].

## 5. Conclusions

We present the first molecular confirmation of a DMV infection in a Cuvier’s BW, although new retrospective studies worldwide could add further evidence of prior cases of infection in this species. The isolate dates from 2002, 10 years after the first Mediterranean epizootic event took place. This is also the second DMV sequence available since then, both of them detected the same year (2002) and collected from stranded cetaceans of different species in the Central East Atlantic Ocean (Canary Islands). These two sequences were identical, indicating that the cross-species infection is not as new as previously suggested.

The epidemic status of the archipelago is that of an endemic situation of several cetacean species rather than an epidemic, although the circulation of strains similar to those later detected in the North-East Atlantic, the Mediterranean and the South-West Pacific indicate that the migration pattern of some of these species could disseminate the virus to other areas and cause epizootic or unusual mortality events.

## Figures and Tables

**Table 1:** Beaked whale specimens included in the present study.

Case Nº	ID Code	Species	Sex	Age	SD	SL	Coordinates	SS	DC	NS	Tested Samples	PCR
1	CET 86	*Z. c.*	F	A	27/11/1999	TNF	28.513067382968398, –16.176165295752952	A	3	G	Skin, lung, liver, kidney	C, F
2	CET 103	*Z. c.*	M	J	19/04/2000	FTV	28.056538132454694, –14.317684331100672	D	3	G	Lung, liver, kidney, brain	C, F
3	CET 108	*Z. c.*	F	A	10/06/2000	TNF	28.195070013507404,–16.42181088596981	D	3	G	Skin, skeletal muscle, lung, liver, kidney	C, F
4	CET 113	*Z. c.*	F	S	16/07/2000	TNF	28.420528319456402, –16.295411555275532	D	3	P	Skin, skeletal muscle	C, F, P
5	CET 134	*M. e.*	F	C	28/06/2001	GC	27.77761716736704, –15.52674193489951	A	1	P	Skin, lung, liver, kidney, brain	C, F, P
6	CET 180	*M.d.*	F	A	24/09/2002	FTV	28.167568704666728, –14.206111760665271	A	2	NE	Skin	C, F, P
7	CET 181	*Z. c.*	M	S	24/09/2002	FTV	28.211852124234827, –14.019063839127336	A	2	NE	Skin, skeletal muscle, lung, mediastinal and mesenteric lymph node, liver, kidney, brain, spleen	C, F
8	CET 182	*Z. c.*	M	S	24/09/2002	FTV	28.19146832713967, –14.122994013994195	D	2	NE	Skin, skeletal muscle, lung, liver, mesenteric lymph node, kidney, brain, spleen	C, F, P
9	CET 183	*Z. c.*	M	S	24/09/2002	FTV	28.186564312428384, –14.257397680615142	D	2	NE	Skin, skeletal muscle, lung, liver, mesenteric lymph node, kidney, brain	C, F, P
10	CET 184	*Z. c.*	M	S	24/09/2002	FTV	28.175274186060737, –14.183223454614003	D	2	NE	Skin, skeletal muscle, lung, liver, mediastinal and mesenteric lymph node, kidney, brain, spleen, thyroid	C, F, P
11	CET 185	*M. e.*	F	A	24/09/2002	FTV	28.638713939591398, –13.830348964499422	D	2	NE	Skin, skeletal muscle, lung, liver, mesenteric lymph node, kidney, brain, spleen	C, F, P
12	CET 189	*Z. c.*	F	A	27/09/2002	FTV	28.63498925367787, –14.026753961358423	D	4	NE	Skin, lung, liver, kidney	C, F, P
13	CET 213	*M. d.*	F	A	28/06/2003	GC	27.933119723146724, –15.37973786995963	A	1	P	Skin, skeletal muscle, lung, liver, kidney, brain	C, F
14	CET 236	*Z. c.*	F	C	21/03/2004	LGr	29.23649579711158, –13.538613817871068	D	3	G	Skin, skeletal muscle, lung, liver, kidney, brain	C, F
15	CET 243	*M. d.*	M	A	18/04/2004	TNF	28.040710105511895, –16.5426071324846	A	1	P	Skin, skeletal muscle, lung, liver, kidney	C, F
16	CET 259	*M. e.*	F	J	21/06/2004	FTV	28.164953252754323, –14.212321787452456	D	2	P	Skin, skeletal muscle, lung, liver, kidney, brain	C, F, P
17	CET 264	*Z. c.*	F	ND	23/07/2004	FTV	28.40069477695754, –13.852448567548068	D	4	G	Liver, skeletal muscle, lung, kidney	C, F
18	CET 265	*Z. c.*	M	A	24/07/2004	FTV	28.744473643139884, –13.940991510227075	D	4	G	Skin, skeletal muscle, lung, liver, kidney	C, F
19	CET 294	*Z. c.*	F	A	18/04/2005	FTV	28.228156055155715, –13.949995729432887	D	4	G	Skin, skeletal muscle, lung, liver, spleen	C, F
20	CET 304	*Z. c.*	F	C	13/07/2005	FTV	28.38034460360221, –14.161693075458741	D	2	P	Skin, skeletal muscle, lung, liver, kidney	C, F
21	CET 322	*Z. c.*	M	A	17/02/2006	GC	27.870618611481834, –15.38656422608156	D	4	I	Skin, lung, liver, kidney	C, F
22	CET 333	*M. e.*	F	S	28/03/2006	EH	29.06227107414346, –13.774638588905537	A	2	G	Skin, skeletal muscle, lung, thymus, liver, mesenteric lymph node, kidney, brain, spleen	C, F
23	CET 334	*M. e.*	F	S	28/03/2006	EH	27.755578877787087, –18.09553359589475	A	2	G	Skin, skeletal muscle, lung, liver, kidney, brain, spleen	C, F
24	CET 338	*M. e.*	F	J	06/04/2006	GC	28.14436864892595, –15.581793297520823	D	2	P	Skin, skeletal muscle, lung, liver, blood, mesenteric lymph node, kidney, brain, spleen	C, F
25	CET 352	*Z. c.*	ND	J	06/07/2006	TNF	28.396221323032027,–16.648897697794496	D	3	P	Skin, lung, liver, kidney, brain, spleen	P
26	CET 354	*M. e.*	M	C	28/07/2006	TNF	28.040710105511895, –16.5426071324846	D	4	M	Skin, lung, liver, kidney, spleen	P
27	CET 379	*M. b.*	M	A	16/04/2007	LZ	28.842800242252817, –13.788144917745738	D	2	E	Skin, lung, liver, kidney, brain, spleen	P
28	CET 471	*Z. c.*	F	S	06/11/2008	FTV	28.607731990351333, –13.8297794478201	D	2	G	Lung, kidney, brain, spleen	P
29	CET 503	*Z. c.*	F	A	21/09/2009	GC	27.750297975375215, –15.568322782893084	D	4	I	Skin, lung, kidney, liver	P
30	CET 510	*M. e.*	M	A	14/12/2009	LZ	29.11583432185095, –13.560196813234894	D	2	E	Skin, lung, liver, mesenteric lymph node, kidney, brain	P
31	CET 547	*M. e.*	M	A	29/08/2010	FTV	28.667401632579352, –14.011239799734405	D	4	M	Skin, lung, liver, mesenteric lymph node, kidney, brain	P
32	CET 576	*Z. c.*	F	A	16/05/2011	LZ	28.84666379719735, –13.788277756973725	D	2	G	Lung, kidney, brain, spleen	F, P
33	CET 579	*Z. c.*	M	S	13/06/2011	TNF	28.031868453191404, –16.575344550886005	D	4	M	Skin, lung, mesenteric lymph node, liver, kidney, brain, spleen	P
34	CET 591	*Z. c.*	F	A	01/11/2011	TNF	28.506326419217828, –16.425276036108105	D	4	I	Skin, lung, prescapular lymph node, liver, kidney, brain, spleen	P
35	CET 593	*Z. c.*	M	A	18/11/2011	GC	27.75629856377708, –15.567180176416969	D	4	G	Skin, lung, prescapular lymph node, liver, kidney	P
36	CET 620	*Z. c.*	M	A	20/05/2012	GC	27.817154801714093, –15.764000859185387	D	4	NE	Skin, lung, liver, kidney, spleen	P
37	CET 624	*Z. c.*	F	A	13/07/2012	LGr	29.286035258319366, –13.481903003501081	D	3	G	Skin, lung, liver, mesenteric lymph node, kidney, brain	F, P
38	CET 631	*M. e.*	M	A	21/10/2012	FTV	28.210771928688324, –14.009197485266776	D	4	G	Skin, lung, penis, palate, esophagus, brain	C, P
39	CET 636	*M. m.*	M	S	30/11/2012	EH	27.765920776532404,–17.910161998928636	D	2	M	Skin, lung, liver, mesenteric lymph node, kidney, brain, spleen	P
40	CET 645	*Z. c.*	M	J	09/02/2013	LZ	29.138139438489326, –13.528173725646699	D	4	M	Skin, liver, brain	F, P
41	CET 680	*Z. c.*	F	N	02/07/2013	GC	27.91040435902637,–15.386896660597422	D	4	M	Skin, lung, liver, intestine, mesenteric lymph node, kidney, brain, spleen	P
42	CET 688	*Z. c.*	F	A	18/11/2013	GC	28.14483414396133, –15.57739880000554	D	4	I	Skin, brain	P
43	CET 695	*M. d.*	F	A	12/07/2014	LZ	29.064020665490503, –13.460099910240649	D	4	M	Skin, lung, liver, mesenteric lymph node, kidney, brain, spleen	P
44	CET 711	*M. d.*	M	S	03/04/2014	EH	27.66937590944239, –18.027604222360484	D	5	P	Skin, lung, liver, mesenteric lymph node, kidney, brain, spleen	P
45	CET 712	*Z. c.*	F	S	28/04/2014	FTV	28.43722859059096, –13.862160820030729	D	4	G	Skin, prescapular lymph node, spleen	P
46	CET 719	*Z. c.*	F	A	06/06/2014	LZ	28.958846751180907, –13.542006915443775	D	3	M	Skin, lung, liver, mesenteric lymph node, kidney, spleen	P
47	CET 720	*Z. c.*	ND	S	10/06/2014	FTV	28.59159662129131,–13.82462012207941	D	4	I	Skin, lung, mesenteric lymph node, liver, kidney, brain	P
48	CET 770	*Z. c.*	M	S	28/07/2015	TNF	28.03511014479708, –16.709505678909764	D	3	G	Skin, lung, liver, intestine, mesenteric lymph node, brain, spleen	P
49	CET 771	*Z. c.*	F	A	05/08/2015	TNF	28.3461443622214, –16.368973513719958	D	2	G	Skin, lung, liver, intestine, mesenteric lymph node, kidney, brain, spleen	P
50	CET 818	*Z. c.*	M	S	16/08/2016	GC	27.756233743172498,–15.67996962889147	D	4	I	Lung, intestine, mesenteric lymph node, kidney, brain, spleen	P
51	CET 824	*M. d.*	F	A	11/11/2016	FTV	28.4768803133933, –13.867047314550653	D	2	E	Skin, prescapular lymph node, liver, kidney, brain, spleen	P
52	CET 827	*M. b.*	F	A	07/12/2016	LG	28.182564817917488, –17.184569356359297	A	4	I	Skin, lung, liver, mesenteric lymph node, intestine, brain, spleen	P
53	CET 833	*Z. c.*	ND	ND	13/02/2017	TNF	28.375804156993976, –16.762765447612193	D	4	I	Skin, lung, liver, mesenteric lymph node, kidney, brain	P
54	CET 852	*M. d.*	F	A	05/05/2017	FTV	28.635507152083715, –14.026812972482025	D	2	P	Skin, lung, liver, mesenteric lymph node, kidney, brain, spleen	P
55	CET 855	*Z. c.*	M	A	22/05/2017	GC	27.734397352882862, –15.622884576151774	D	3	I	Skin, lung, liver, intestine, mesenteric lymph node, kidney, brain, spleen	P

Notes: *Z. c.: Ziphius cavirostris; M. d.: Mesoplodon densirostris; M. e.: Mesoplodon europaeus; M. b.: Mesoplodon bidens; M. m.: Mesoplodon mirus*. SD: stranding date; SL: stranding location (GC: Gran Canaria; TNF: Tenerife; FTV: Fuerteventura; LZ: Lanzarote; LG: La Gomera; EH: El Hierro; LGr: La Graciosa); SS: stranding stage; DC: decomposition stage (1 = extremely fresh carcass; 2 = fresh carcass; 3 = moderate decomposition; 4 = advanced decomposition; 5 = mummified or skeletal remains); NS: nutritional status (G: good; M: moderate; P: poor; E: emaciated; NE: not evaluated; I: indeterminate); Sex: F = female; M = male; ND = not determined; age: A = adult; S = subadult; J = juvenile; C = calf, *n* = neonate. PCR: polymerase chain reaction (C: conventional one-step RT PCR [42,43,44]; F: one step RT-qPCR (F gene) [45]; P: pan RT-qPCR [46].

**Table 2:** Summary of the percentages of cetacean morbillivirus prevalences reported in other species, geographical zones and dates.

Region	Period	Species	Diagnostic Test	% Prevalence	References
Portuguese and Galician coasts (North-East Atlantic Ocean)	2004–2015	*Stenella coeruleoalba*, *Delphinus delphis*	PCR	5.7% (16/279)	[64]
Southeastern coast of USA (North-West Atlantic Ocean)	2003–2007	*Tursiops truncatus*	Serology	9.8% (12/122)	[65]
The Canary Islands (Central East Atlantic Ocean)	2003–2015	*Grampus griseus*	PCR	16.6% (2/12)	[66]
Northern Gulf of Mexico and USA (West Atlantic Ocean)	2010–2014	*Tursiops truncatus*	PCR	9.9% (14/142) stranded cetaceans; 1% (1/83) free-ranging live	[67]
			Serology	29% (5/7) of live stranded 23% (23/102) of live free-ranging	
			Histopathology + PCR	6.6% (9/136)	
Italy (Mediterranean Sea)	2006–2014	*Balaenoptera physalus*	PCR Serology IHC	21.74% (5/23)	[68]
Hawaii (Pacific Ocean)	1997–2014	*Megaptera novaeangliae*, *Kogia breviceps*, *Stenella longirostris*, *Ziphius cavirostris*, *Stenella frontalis*, *Indopacetus pacificus*, *Stenella coeruleoalba*, *Mesoplodon densirostris*, *Tursiops truncatus*, *Physeter macrocephalus*, *Steno bredanensis*, *Grampus griseus*	PCR	24% (15/62)	[13]
Italy (Mediterranean Sea)	1998–2014	*Stenella coeruleoalba*, *Tursiops truncatus*, *Balaenoptera physalus* and *Globicephala melas*	Serology	32.8% (23/70)	[69]
Central California coast (Pacific Ocean)	2000–2015	*Phocoena phocoena*, *Delphinus capensis*, *Lagenorhynchus obliquidens*, *Stenella coeruleoalba*, *Grampus griseus*	PCR (1/11)	36.36% (4/11)	[70]
			Serology (3/11)		
Brazil (South Atlantic Ocean)	2010–2017	*Eubalaena australis*	PCR	37.5% (3/8)	[71]
Australia (Pacific Ocean)	2005–2011	*Peponocephala electra*, *Tursiops aduncus*, *Lagenodelphis hosei*, *Tursiops truncatus*, *Balaenoptera edeni*	Serology	48.1% (13/27)	[72]

## Data Availability

The DMV obtained sequences from a Cuvier’s beaked whale have been deposited in GenBank (under accession numbers: OM055653, OM055654).
